# Transient Inactivation of the Medial Prefrontal Cortex and Ventral Hippocampus Impairs Active Place Avoidance Retrieval on a Rotating Arena

**DOI:** 10.3389/fncir.2021.634533

**Published:** 2021-04-28

**Authors:** Daniela Cernotova, Ales Stuchlik, Jan Svoboda

**Affiliations:** Laboratory of the Neurophysiology of Memory, Institute of Physiology of the Czech Academy of Sciences, Prague, Czechia

**Keywords:** spatial memory, muscimol, hippocampo-prefrontal pathway, active place avoidance, rotating arena

## Abstract

It is well known that communication between the medial prefrontal cortex (mPFC) and the ventral hippocampus (vHPC) is critical for various cognitive and behavioral functions. However, the exact role of these structures in spatial coordination remains to be clarified. Here we sought to determine the involvement of the mPFC and the vHPC in the spatial retrieval of a previously learned active place avoidance task in adult male Long-Evans rats, using a combination of unilateral and bilateral local muscimol inactivations. Moreover, we tested the role of the vHPC-mPFC pathway by performing combined ipsilateral and contralateral inactivations. Our results showed not only bilateral inactivations of either structure, but also the combined inactivations impaired the retrieval of spatial memory, whereas unilateral one-structure inactivations did not yield any effect. Remarkably, muscimol injections in combined groups exerted similar deficits, regardless of whether the inactivations were contralateral or ipsilateral. These findings confirm the importance of these structures in spatial cognition and emphasize the importance of the intact functioning of the vHPC-mPFC pathway.

## Introduction

The encoding, consolidation, and retrieval of spatial memories have long been associated with structures of the medial temporal lobe, especially the hippocampal formation. Traditionally, spatial functions have been associated with the dorsal hippocampus (dHPC; Moser et al., 1993; Pothuizen et al., 2004). In agreement with functional dissociation in the dorsoventral axis of the hippocampus (Bannerman et al., 2004), the role of the vHPC has been classically ascribed mainly to anxiety and emotionality (Bannerman et al., 2003), locomotion (Bast et al., 2001), and the response to stress (Herman et al., 1992). Spatial processing in the vHPC, however, remains a subject of discussion. Studies using partial lesions to the vHPC in rats did not impair learning performance in the Morris water maze (MWM) until the lesion impacted the whole vHPC (in comparison, the dHPC showed impairments already from lesions of 20%, and increased with a higher volume lesioned; Moser et al., 1993, 1995). Other studies have reported that the vHPC is not involved in spatial reference and working memories (Bannerman et al., 2003; Pothuizen et al., 2004; Potvin et al., 2006). However, in de Hoz et al. (2003) modified the experimental protocol used by Moser et al. (1995), and showed that the vHPC affected spatial learning equally to the dHPC if the training protocol was slightly modified (4 trials a day for 8 days, compared to previous 8 trials a day for 6 days). Subsequent studies have confirmed that lesions to the vHPC do impair spatial learning (Ferbinteanu et al., 2003) and spatial memory retrieval (Broadbent et al., 2004; Loureiro et al., 2012). Lidocaine inactivations of the vHPC have shown impairments in spatial memory retrieval as well (Floresco et al., 1997; Seamans et al., 1998).

A more recent study investigated intercommunication between the dHPC and the vHPC during the MWM task learning in rats (Lee et al., 2019). In this study, the activity of the hippocampi was locally inhibited by muscimol in bilateral or combined inactivations. It was found that spatial performance was also significantly impaired when silencing the whole hippocampi, and similarly impaired in bilateral dHPC and the vHPC inactivations. Ipsilateral inactivations (in one hemisphere) showed only minor impairment, but inactivation of one dHPC and the contralateral vHPC resulted in remarkable deficits, resembling bilateral inactivations (Lee et al., 2019). Overall, despite the initial opinion that the vHPC is not involved in spatial learning, newer studies have shown that the vHPC is indeed needed for spatial learning and the representation of spatial memories, and together with the above-mentioned findings, it can be concluded that the whole hippocampus, acting as a unitary structure, is needed for spatial processing.

The roles of various parts of the prefrontal cortex in spatial memory retrieval are also the subject of debates. The PFC is further divided into functionally distinct segments that mediate different forms of flexible behavior. In rats, possibly the most important region is the mPFC, which is further divided into three subdivisions, from the ventral to the dorsal direction: the infralimbic cortex (IL), the prelimbic cortex (PL), and the anterior cingulate (ACC) (Hamilton and Brigman, 2015). A number of studies have shown that lesioning or inactivating the mPFC pharmacologically in rats impairs the attentional set-shift between strategies, sets, or rules, without impairing reversal learning (for example Ragozzino et al., 1999; Birrell and Brown, 2000; Boulougouris et al., 2007; Floresco et al., 2008; Malá et al., 2015).

Regarding the retrieval of spatial memory, the study by Jo et al. (2007) showed that mPFC is crucial for this function when tested in the Morris water maze (MWM) under only a partial availability of cues, but not in full-cue conditions. Other support for the involvement of the PFC in spatial and non-spatial memory retrieval has come from the studies by Leon et al. (2010), who showed that the retrieval of spatial remote memory in the MWM is affected by the intra mPFC application of an inhibitor of the mitogen-activated protein kinase (MAPK)/ERK pathway, and by Churchwell et al. (2010) who used a Hebb-Williams maze to show that lidocaine inactivation of the mPFC, but not orbital frontal cortex, impaired both the acquisition and retrieval of memory. Moreover, Cholvin et al. (2016) reported that both mPFC and dHPC contribute to the retrieval of recent spatial memory.

The vHPC and mPFC are known to interact with intensive frontotemporal crosstalk that supports spatial cognition (Jones and Wilson, 2005; Wang and Cai, 2006) and are significantly impaired in several neuropsychiatric conditions such as schizophrenia (Adams et al., 2020). To further evaluate the precise roles of the vHPC and mPFC and their interactions, we sought to determine the effects of single or combined local inactivations of these structures in the retrieval of an aversive spatial memory test, the active place avoidance on a rotating arena (Carousel; Bures et al., 1997; Fenton et al., 1998), for review see Stuchlík et al. (2013). The hypothesis was that both separate and combined switching-off of the vHPC and mPFC would impair the retrieval of spatial memory in this dry-arena paradigm.

## Materials and Methods

Forty-nine male Long-Evans rats (3–4 months old, breeding core of the Institute of Physiology of the Czech Academy of Sciences) were included in the statistical analysis. The animals were housed in pairs and kept at a 12/12 h light/dark cycle. The rats were randomly assigned into unilateral (UNI) vHPC (*n* = 8) and mPFC (*n* = 8), bilateral (BI) vHPC (*n* = 9) and mPFC (*n* = 10) or contralateral (CONTRA; *n* = 7) and ipsilateral (IPSI; *n* = 7) vHPC-mPFC groups. Because the vHPC-mPFC connections are mainly ipsilateral (Jay et al., 1989; Hoover and Vertes, 2007), we assumed the contralateral inactivations disconnected functional pathways in both hemispheres. The inactivated vHPC could not transfer information to the mPFC on one side, while the attenuated activity in the mPFC on the other side could not process this information. Ipsilateral inactivations disconnected the information transfer in one hemisphere only; the other hemisphere could compensate for the impairment. The animals were food-restricted and maintained at 85–90% of their body weight. A day before the first behavioral training, a needle was pierced through a skin fold between shoulders to allow shock delivery throughout sessions, with the tip bent to prevent slipping out. This procedure was painless and did not require anesthesia. The experiment was done during the light phase of the day. All animal treatment complied with the Animal Protection Code of the Czech Republic and the European Community Council directive (2010/63/EC).

Rats were anesthetized with continuous-flow isoflurane (5% for induction, 2–2.5% for maintenance) and mounted in a stereotaxic apparatus. Custom-made guiding cannulas (22-gauge, 11 mm in length) were implanted to the brain, relative to *bregma*, aiming at the mPFC (+3.2 AP and ±1.3 ML, a 10° angle from the midline, 3 mm below the skull), or the vHPC (−5.2 AP and ±5.4 ML, 6 mm below the skull), depending on the group. Dummy cannulas were inserted into the guiding cannulas and remained there until the injections were initiated. Anchoring screws were mounted to the skull, and, together with the cannulas, embedded with dental cement. Local antiseptic and anesthetic were applied on the sutured wound, and postoperative care was provided by adding antibiotics and analgesics to the water. The rats were checked daily and left to recover for 14 days.

**FIGURE 1:**
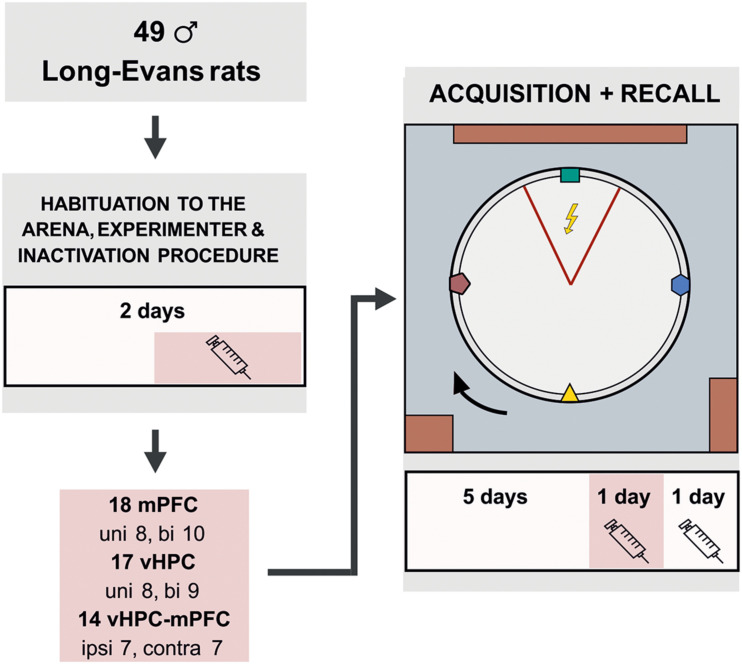
Experimental design for the active place avoidance learning and retrieval. The rats were habituated on the arena for 2 days prior to the training. The experimental task consisted of 5 days of 20 min learning sessions, in which the rats learned to avoid a static sector on the rotating arena based on room-bound cues. The colored cues, placed on the arena wall and rotating with the arena, were irrelevant for successful avoidance. On day 2 of habituation and on the day 6 (20 min prior to the training), the rats were given muscimol injections based on their experimental group. The session on day 7 was used as a control; saline injections were administered to the rats 20 min before the session, similarly as in the experimental groups. bi, bilateral; uni, unilateral; ipsi, ipsilateral; contra, contralateral.

A GABA_*A*_ agonist muscimol (1 μg/μl diluted in 0.9% saline, stored at −20°C; Sigma-Aldrich) was used to temporarily inactivate neurons (Martin, 1991). Muscimol or sterile saline were injected slowly (0.5 μl/side, 1 min duration) into the appropriate structures with a 5 μl Hamilton syringe, connected by a polyethylene tube with an injection cannula (27-gauge, 12 mm in length). The injection cannula was removed after another 30 secs to avoid reflux of the solution. Dummy cannulas were placed back inside the guiding cannulas when the microinjection was completed. One injection of muscimol or saline was administered to the rats 1 day before the onset of behavioral training for habituation to the procedure or drug exposure. Other injections were given 20 min prior to the day 6 and 7 sessions (design of the study is illustrated in Figure 1).

Rats were trained to actively avoid an unmarked 60° sector on a rotating arena (1 revolution per minute); the apparatus was previously described in detail by Svoboda et al. (2015a, b). A camera monitored the position of the rat relative to the location of two LEDs, one on the outer edge of the arena, the other attached to a harness on the rat’s back. Commercially available Tracker software (Biosignal Group, United States) was located in an adjacent operating room and used to record the location and deliver mild shocks (50 Hz) to the rat if it entered the forbidden sector for more than 300 ms. The shocks were repeated every 500 ms, until the rat left the sector for more than 300 ms. The intensity of shocks ranged from 0.3 to 0.6 mA, depending on the rat’s sensitivity to the shocks. The current was adjusted individually for each rat to elicit a quick escape reaction, but prevent freezing. The shocks were delivered to a subcutaneous needle on animals back through grounded floor from a custom-based source with constant current regulation located in the adjacent room (a defined current was given despite the differences in resistance). The position of the sector was defined by extramaze cues (e.g., table, cabinet, door). At the beginning of each session, a rat was placed opposite the shock sector. Then an experimenter had to move to the adjacent room to start computer tracking, resulting in a lag of around 3–5 s. The initial configuration of the room and arena cues was the same for all animals.

Prior to the training, the animals were given 2 days of handling and 5 min of arena acclimation (no shocks or rotation) with grains being dispersed on the arena to avoid hyponeophagia. Since locomotion was essential for successful avoidance, collecting grains encouraged active movement during the experiment. The initial task acquisition followed (arena rotating, the to-be-avoided sector in a static position), consisting of 5 sessions (20 min/day). Two more sessions tested the spatial retrieval, with muscimol injections on day 6 and saline injections on day 7.

**FIGURE 2:**
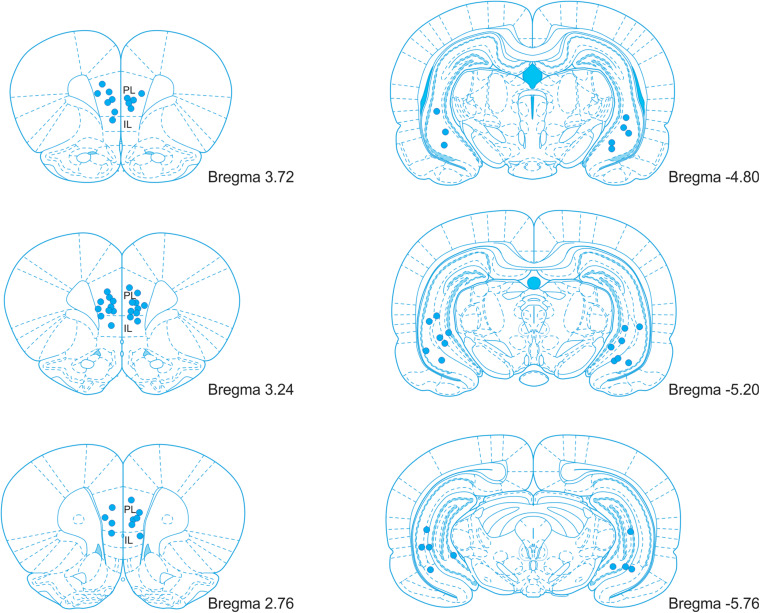
An illustration of the cannula placement locations in the mPFC and vHPC. PL, prelimbic cortex; IL, infralimbic cortex. Adopted from Paxinos and Watson (2007).

After completing the experiment, the rats were deeply anesthetized, and 1 μl of ink (diluted in PBS) was infused through each cannula by an infusion pump (200 nl/min) to label the sites of injection. Perfused brains were fixed in 4% paraformaldehyde for 24 hours, soaked in 30% sucrose solution, and stored at −80°C for further processing. The brains were either cut coronally by a blade in the cannula locations to verify the site of ink injection or cut to 50 μm coronal sections using a cryostat. Every fifth section was collected and processed with Nissl staining (0.1% Cresyl Violet stain solution). Cannula placements were verified accordingly to the rat brain atlas (Paxinos and Watson, 2007). Brains with incorrect cannula placement were excluded from the analysis. The placement of the cannula tips is illustrated in Figure 2.

Additionally, six rats with locomotor issues after muscimol application (two from the BI/vHPC, two from the IPSI, and two from the CONTRA groups) and 12 non-learners (six from the BI/vHPC, two from the UNI/vHPC, one from the UNI/mPFC, one from the IPSI, and two from the CONTRA groups) were excluded from the statistical analysis. Five implants in the BI/vHPC group became detached during the experiment; therefore, these rats were sacrificed immediately. One rat from the CONTRA group was excluded because of inaccurate cannula placement.

For the evaluation of muscimol inactivation effect on retrieval, we compared performance on days 6 (muscimol injections), and 7 (saline injections) in several parameters, namely the total path, the time to the first entrance, the number of entrances, and shocks. Skewed data were logarithmically transformed to meet the normal distribution (Table 1), and a two-way ANOVA with repeated measures on sessions was used to analyze these parameters (SESSION × GROUP). One rat from the UNI/vHPC group was identified as an outlier and was excluded from the analysis of entrances and shocks. These parameters for the vHPC groups were analyzed using a mixed-effects model. The day 6 session was split into 5 min intervals and the number of entrances, shocks, and the total path were analyzed by a two-way ANOVA with repeated measures on time intervals (TIME × GROUP). When needed, Sidak’s multiple comparison *post hoc* test was used to compare performance within the individual sessions or time intervals. The level of significance was at α < 0.05. Measured data were analyzed by the open-source program Carousel Maze Manager (v.0.4.0.) (Bahník, 2014) and statistical analysis was performed using GraphPad Prism 8.0.1. Data for days 6 and 7 are shown as individual data ± SD, graphs showing 5 min comparisons on day 6 and Supplementary Data from acquisition are depicted as group means ± SD.

**TABLE 1:** Summary of two-way ANOVA results between the experimental groups on days 6 and 7.

**(A) Unilateral/bilateral mPFC**				

**(1) Sessions on days 6 and 7**	**Effect of session**	**Effect of group**	**Effect of interaction**	**Transformation**
Entrances	***F*(1, 16) = 24.81, *p* = 0.0001**	*F*(1, 16) = 1.729, *p* = 0.2071	*F*(1, 16) = 2.715, *p* = 0.1189	–
Shocks	***F*(1, 16) = 36.25, *p* < 0.0001**	*F*(1, 16) = 0.4276, *p* = 0.5225	*F*(1, 16) = 2.098, *p* = 0.1668	ln(y + 1)
Time to the first entrance	***F*(1, 16) = 17.43, *p* = 0.0007**	*F*(1, 16) = 0.1619, *p* = 0.6927	*F*(1, 16) = 0.03751, *p* = 0.8489	–
Total path	*F*(1, 16) = 0.05427, *p* = 0.8187	***F*(1, 16) = 14.28, *p* = 0.0016**	*F*(1, 16) = 2.459, *p* = 0.1364	ln(y − 35)

**(2) 5-min intervals on day 6**	**Effect of time**	**Effect of group**	**Effect of interaction**	**Transformation**

Entrances	*F*(3, 48) = 2.765, *p* = 0.0519	*F*(1, 16) = 3.659, *p* = 0.0738	*F*(3, 48) = 0.2237, *p* = 0.8795	ln(y + 1)
Shocks	***F*(3, 48) = 3.231, *p* = 0.0304**	*F*(1, 16) = 2.566, *p* = 0.1288	*F*(3, 48) = 1.108, *p* = 0.3549	ln(y + 1)
Total path	***F*(3, 48) = 0.6182, *p* = 0.0016**	***F*(1, 16) = 6.177, *p* = 0.0244**	*F*(3, 48) = 0.6182, *p* = 0.6066	ln(y − 8)

**(B) Unilateral/bilateral vHPC**				

**(1) Sessions on days 6 and 7**	**Effect of session**	**Effect of group**	**Effect of interaction**	**Transformation**

Entrances	***F*(1, 14) = 16.15, *p* = 0.0013**	*F*(1, 15) = 2.435, *p* = 0.1395	*F*(1, 14) = 3.496, *p* = 0.0826	ln(y + 1)
Shocks	***F*(1, 14) = 15.38, *p* = 0.0015**	*F*(1, 15) = 3.203, *p* = 0.0937	*F*(1, 14) = 4.341, *p* = 0.056	ln(y + 1)
Time to the first entrance	*F*(1, 15) = 1.31, *p* = 0.2704	*F*(1, 15) = 0.6726, *p* = 0.425	*F*(1, 15) = 0.05935, *p* = 0.8108	ln(y + 1)
Total path	*F*(1, 15) = 0.0129, *p* = 0.9111	*F*(1, 15) = 0.2139, *p* = 0.6504	*F*(1, 15) = 0.001971, *p* = 0.9652	–

**(2) 5-min intervals on day 6**	**Effect of time**	**Effect of group**	**Effect of interaction**	**Transformation**

Entrances	***F*(3, 42) = 7.067, *p* = 0.0006**	***F*(1, 14) = 4.684, *p* = 0.0482**	*F*(3, 48) = 0.2237, *p* = 0.6191	ln(y + 1)
Shocks	***F*(3, 42) = 5.653, *p* = 0.0024**	*F*(1, 14) = 4.377, *p* = 0.0551	*F*(3, 42) = 1.321, *p* = 0.2802	ln(y + 1)
Total path	***F*(3, 45) = 19.11, *p* < 0.0001**	*F*(1, 15) = 0.1368, *p* = 0.7167	*F*(3, 45) = 0.05478, *p* = 0.9829	–

**(C) Contralateral/ipsilateral mPFC-vHPC**				

**(1) Sessions on days 6 and 7**	**Effect of session**	**Effect of group**	**Effect of interaction**	**Transformation**

Entrances	***F*(1, 12) = 26.13, *p* = 0.0003**	*F*(1, 12) = 0.03442, *p* = 0.8559	*F*(1, 12) = 0.02016, *p* = 0.8894	–
Shocks	***F*(1, 12) = 30.74, *p* = 0.0001**	*F*(1, 12) = 4.28e-005, *p* = 0.9949	*F*(1, 12) = 0.00716, *p* = 0.934	ln(y + 1)
Time to the first entrance	*F*(1, 12) = 0.5818, *p* = 0.4603	***F*(1, 2) = 5.35, *p* = 0.0393**	*F*(1, 12) = 0.9045, *p* = 0.3603	ln(y + 1)
Total path	***F*(1, 12) = 6.534, *p* = 0.0252**	*F*(1, 12) = 0.6342, *p* = 0.4413	*F*(1, 12) = 0.1975, *p* = 0.6647	–

**(2) 5-min intervals on day 6**	**Effect of time**	**Effect of group**	**Effect of interaction**	**Transformation**

Entrances	***F*(3, 36) = 3.057, *p* = 0.0406**	*F*(1, 12) = 0.04412, *p* = 0.8372	*F*(3, 36) = 0.5988, *p* = 0.62	–
Shocks	***F*(3, 36) = 7.051, *p* = 0.0008**	*F*(1, 12) = 0.009952, *p* = 0.9222	*F*(3, 36) = 0.3766, *p* = 0.7704	ln(y + 1)
Total path	***F*(3, 36) = 3.811, *p* = 0.018**	*F*(1, 12) = 0.548, *p* = 0.4734	*F*(3, 36) = 0.4077, *p* = 0.7484	–

*Significant values are in bold.*

## Results

Only the animals that had learnt the initial place avoidance task were included in the statistical analysis. Data of 1 rat in the CONTRA group on day 5 were lost due to technical issues. Therefore, we used a mixed-effects model to analyze performance of the CONTRA and IPSI groups during the acquisition. Throughout the acquisition, rats reduced the number of entrances and shocks and prolonged the time to the first entrance (Supplementary Figures 1A–C), as confirmed by significant effects of session in these parameters (Supplementary Table 1). The group and interaction effects were non-significant. Two-way ANOVA results for the total path in the vHPC and combined groups were non-significant in all main and interaction effects, however, a significant effect of session and group in the mPFC group was revealed. *Post hoc* tests revealed differences between groups on days 4 and 5 (Supplementary Figure 1D).

**FIGURE 3:**
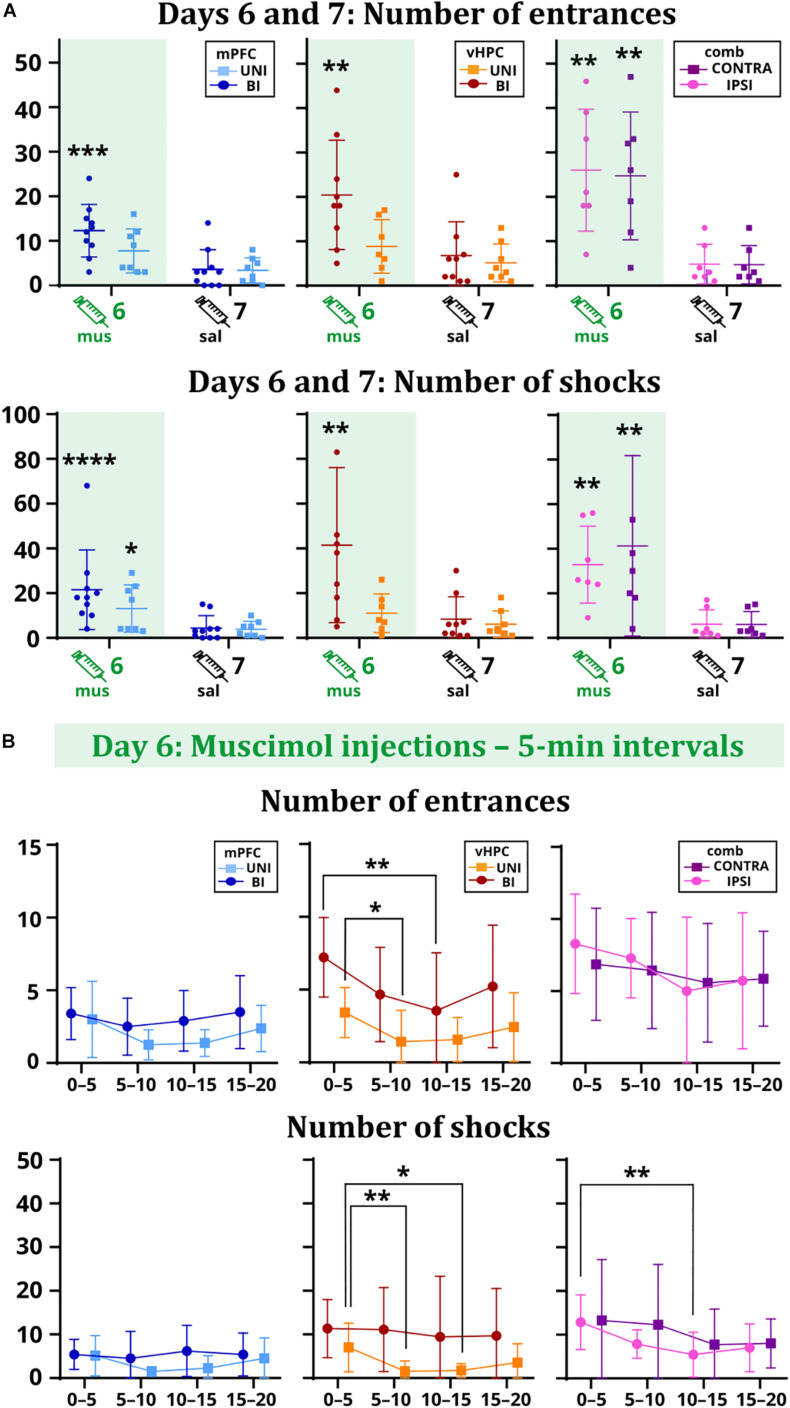
Performance of the rats on days 6 vs. 7 **(A)** and within the day 6 session **(B). (A)** The BI/mPFC group increased the number of entrances (*p* = 0.0003) and shocks (*p* < 0.0001) on the inactivation day. The number of shocks increased in the UNI/mPFC group as well (*p* = 0.0147). For the vHPC groups, only the bilaterally inactivated rats showed significant problems with not entering the sector (*p* = 0.0012) and received a higher amount of shocks (*p* = 0.001). Increased numbers of entrances (IPSI: *p* = 0.0059, CONTRA: *p* = 0.0085) and shocks (IPSI: *p* = 0.0036, CONTRA: *p* = 0.0045) were observed for both combined groups. **(B)** The performance of the mPFC groups remained stable over time. Both vHPC groups significantly reduced the number of entrances (*p* = 0.0202 for intervals 0–5 vs. 5–10 for the UNI/vHPC group; *p* = 0.0069 for intervals 0–5 vs. 10–15 for the BI/vHPC group), but only the UNI/vHPC group had a markedly reduced number of shocks (*p* = 0.0061 for intervals 0–5 vs. 5–10; *p* = 0.0326 for intervals 0–5 vs. 10–15). In the combined groups, the number of entrances remained high during the whole session; however, the number of shocks gradually decreased, significantly in the case of the IPSI group between the intervals 0–5 vs. 10–15 (*p* = 0.0034). *p < 0.05; **p < 0.01; ***p < 0.001; ****p ≤ 0.0001.

All results for main and interaction effects on days 6 and 7 are summarized in Table 1. Impaired avoidance was observed in muscimol-treated animals, with significant effects of session on the number of entrances and shocks. *Post hoc* tests confirmed this effect only in the case of the BI/mPFC, BI/vHPC, and both combined groups. Moreover, a higher number of shocks was found in the UNI/mPFC group as well (Figure 3A). Effects of group and session × group interaction in these parameters were non-significant. The main effects of time on day 6 indicate the groups manifested intra-session learning and could decrease the number of entrances and shocks within the inactivation session, with a significant group effect on entrances in the vHPC groups. *Post hoc* tests revealed that the BI/vHPC group had consistently more entrances than the UNI/vHPC group; however, they both displayed a significant decrease in the number of entrances over time compared to all other groups (Figure 3B). In contrast, only the UNI/vHPC and IPSI groups lowered the number of shocks, pointing to different patterns of intra-session learning (Figure 3B). All other group and all time × group interaction effects on day 6 were non-significant.

**FIGURE 4:**
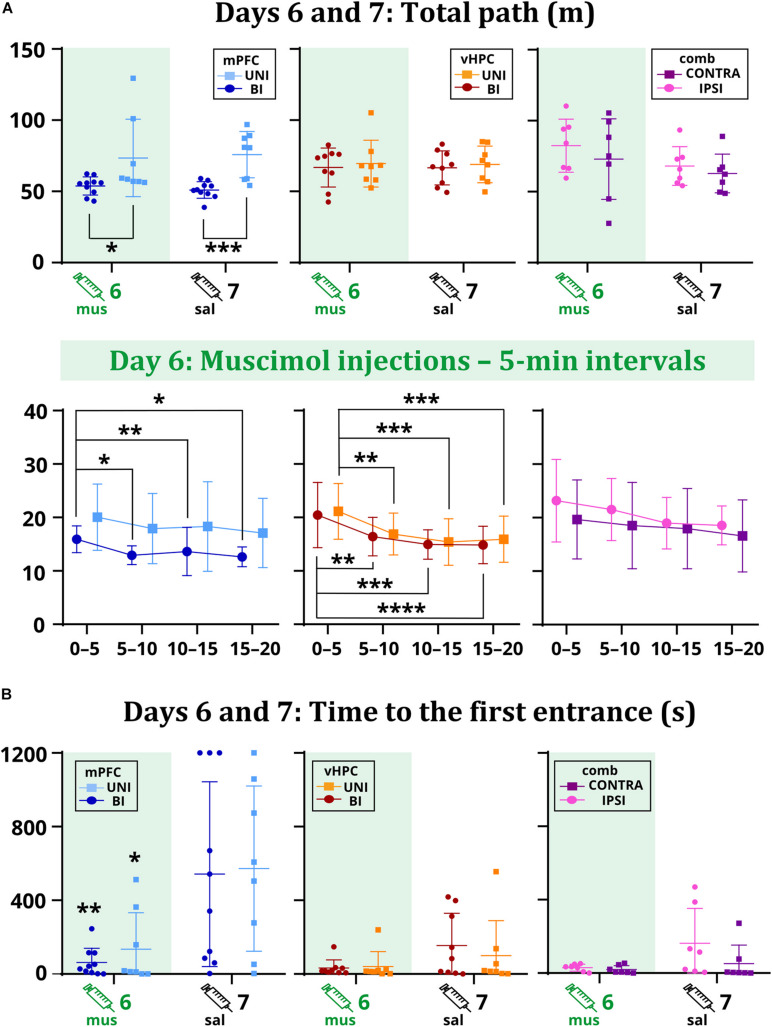
The total path on days 6 and 7 **(A)** and the time to the first entrance on days 6 and 7 **(B)**. Muscimol had no effect on the total path, but differences were observed between the mPFC groups (*p* = 0.0261 on day 6; *p* = 0.0006 on day 7). Analysis of the sixth day showed a tendency to move less for the BI/mPFC group (*p* = 0.0371 between the intervals 0–5 vs. 5–10; *p* = 0.0029 vs. 10–15; *p* = 0.0126 vs. 15–20), but also for the UNI/vHPC (minutes 0–5: *p* = 0.0074 vs. 5–10; *p* = 0.0002 vs. 10–15; *p* = 0.0007 vs. 15–20) and BI/vHPC groups (minutes 0–5: *p* = 0.0067 vs. 5–10; *p* = 0.0001 vs. 10–15; *p* < 0.0001 vs. 15–20). No difference was observed in the combined groups. The first entrance occurred significantly earlier on day 6 than day 7 for the UNI/mPFC (*p* = 0.0095) and BI/mPFC (*p* = 0.0332) groups. *p < 0.05; **p < 0.01; ***p < 0.001; ****p ≤ 0.0001.

Time to the first entrance was not affected by the inactivation in the vHPC and combined groups, but a significant effect of session in the mPFC group suggests that muscimol might have affected memory retrieval. Results show both UNI/mPFC and BI/mPFC groups first entered the shock sector much earlier on day 6 than 7 (Figure 4B). Two-way ANOVA revealed group differences in combined groups, but no effect of session or the session x group interaction. *Post hoc* tests found there was no difference within the groups between sessions. Group or session × group interaction effects in the vHPC groups were non-significant.

As confirmed by the non-significant effects of session and session x group interactions in the mPFC and vHPC groups, muscimol did not affect the total path. Two-way ANOVA found a significant group effect between the mPFC groups. *Post hoc* tests revealed that the BI/mPFC group traveled a shorter distance than the UNI/mPFC group on both sessions (Figure 4A). Analysis of 5 min intervals confirmed the group differences and revealed a significant effect of time within the inactivation session; the BI/mPFC group showed gradually decreasing levels of locomotion (Figure 4A). A similar relationship was found for both vHPC groups, as indicated by a significant effect of time on day 6 (Figure 4A). Significant effects of session and time in combined groups point to variance between and within sessions, respectively, but *post hoc* tests did not confirm any difference between the groups (Figure 4A). Time or session × group interaction effects in combined groups were not significant.

## Discussion

Efficient place avoidance on a rotating arena requires cooperative activity of both dorsal hippocampi to segregate and coordinate irrelevant arena-bound and relevant room-bound cues in meaningful spatial frameworks (Wesierska et al., 2005; Kelemen and Fenton, 2010). Unilateral inactivation of the dorsal hippocampus by tetrodotoxin (TTX) has been found to compromise these processes, manifested by an increased number of entrances into the punished sector (Cimadevilla et al., 2001; Wesierska et al., 2005). Olypher et al. (2006) reported that unilateral TTX injection results in discoordinated neuronal activity in the contralateral hippocampus, preventing rats from creating distinct representations that would reflect dissociated reference frames of the room and arena. While rotation makes the conflict of arena-based and room-based spatial information particularly challenging, place avoidance on a stationary arena still depends on the hippocampus, albeit a deficit is observed only after bilateral inactivation (Kubík et al., 2006). Kubík and Fenton (2005) predicted that on a rotating arena, inactivations would preferentially affect acquisition rather than retrieval. Here we report deficits in the retrieval of avoidance behavior after bilateral, but not unilateral inactivations of the vHPC, and contrary to expectations, rats with a bilaterally inactivated vHPC surprisingly displayed within-session learning in terms of the decreasing number of entrances, indicating some spare capacity for new acquisition. On the other hand, animals did not decrease the number of shocks once they entered the shock zone, indicative of an impaired strategy to avoid multiple shocks. It seems unlikely that this is due to an altered perception of the shock stimulus, even though the vHPC has been linked to responding to aversive unconditioned stimuli (Wang et al., 2015). If this were the case, then the number of entrances into the shock sector would not decrease either. It should be pointed out that bilaterally inactivated vHPC rats showed only a mild within-session improvement, with an even increasing number of entrances in the last 5 min (Figure 3B). Then we cannot rule out an alternative hypothesis that some spared learning was due to incomplete inactivation of vHPC. In summary, these results confirm the importance of the vHPC in spatial avoidance on a rotating arena, but point to the different nature of processes provided by the dHPC and vHPC, respectively.

The role of the medial prefrontal cortex in place avoidance in the Carousel has been recently evaluated by Park et al. (2019). They found excitotoxic lesions of the mPFC exerted no effect on acquisition. This is in contrast to our data showing a clear avoidance deficit in rats with the mPFC bilaterally inactivated. In addition to affecting different memory stages, permanent lesions are known to produce less profound deficits as their function can be compensated with time. In our study, bilateral inactivation of the mPFC not only compromised the retrieval of spatial avoidance but also prevented within-session learning, as evidenced by the constant frequency of entering the sector in the inactivation session. However, it should be pointed out that the total number of entrances (or shocks) was not as high as in the case of bilateral inactivation of the vHPC, so animals did not have as much space to improve. Based on their negative results, Park et al. (2019) concluded that the mPFC does not cooperate with the hippocampus to provide cognitive control, a process underlying efficient place avoidance. Here we observed rather the opposite, lending further support from combinational inactivations. Not only did bilateral disturbance of the hippocampo-prefrontal pathway by contralateral inactivations impair spatial avoidance on the arena, but disturbance of the pathway just in one hemisphere by ipsilateral inactivations also yielded the same effect. However, if one intact hippocampo-prefrontal pathway is not sufficient to support efficient place avoidance, why was a deficit not seen after unilateral inactivations of either the vHPC or mPFC? A corroborative hypothesis may posit that within the network supporting cognitive control, the function of one blocked structure (mPFC or vHPC) in one hemisphere may be compensated for by its counterpart in the other hemisphere and by interhemispheric activity between the spared structures. It is well established that both the vHPC and mPFC have intense commissural projections. Thus, if mPFC is blocked unilaterally, the spared contralateral mPFC compensates for its function, and together with commissural communication of both ventral hippocampi support the cognitive control. The same logic applies for unilateral vHPC inactivation, but cannot work for a blockade of any two sites.

It is noteworthy that early investigations of hippocampal involvement in place avoidance in the Carousel used TTX (Cimadevilla et al., 2001; Kubík and Fenton, 2005; Wesierska et al., 2005), a potent sodium channel blocker that also blocks fibers of passage. Therefore, TTX not only interfered with neuronal activity of the dHPC but also with pathways leading through the fimbria-fornix system, including the vHPC-mPFC direct connection. Therefore, TTX studies might have also inadvertently examined this pathway. Indeed, ipsilateral muscimol inactivations of the vHPC and mPFC resemble deficits seen after the unilateral TTX inactivation of the dHPC.

## Conclusion

Our study establishes the important roles of the mPFC and vHPC in the retrieval of spatial memory, a domain that has been traditionally ascribed to the dHPC. Specifically, bilateral muscimol inactivations of either structure were found to impair parameters related to cognitive control, such as the number of entrances into a punished region, while leaving general locomotor activity unaffected. Furthermore, this work emphasizes hippocampo-prefrontal communication as a critical link in a cognitive control mediating network, because both ipsilateral and contralateral hippocampo-prefrontal inactivations yielded significant deficits in spatial avoidance on the arena. Further investigations are required to shed light on what specific processes vHPC and mPFC perform in this network.

## Data Availability Statement

The raw data supporting the conclusions of this article will be made available by the authors, without undue reservation.

## Ethics Statement

The animal study was reviewed and approved by the Local and Ministry Committee on Animal Protection of Welfare, Projects of Experiments No. 80/2016, 50/2017.

## Author Contributions

AS and JS: conceptualization of the study, design, draft writing, supervision, and funding acquisition. DC and JS: conducting experiments and data analysis. All authors contributed to the article and approved the submitted version.

## Conflict of Interest

The authors declare that the research was conducted in the absence of any commercial or financial relationships that could be construed as a potential conflict of interest.
