# Supplementary material for: Transient Inactivation of the Medial Prefrontal Cortex and Ventral Hippocampus Impairs Active Place Avoidance Retrieval on a Rotating Arena
Source: Front Neural Circuits. 2021 Apr 28;15:634533. doi: 10.3389/fncir.2021.634533 (PMC8113689; doi:10.3389/fncir.2021.634533)
Supplement: Supplementary file 1 [file Data_Sheet_1.docx]

Supplementary Material

# Supplementary Figures and Tables

## Supplementary Figure

**Supplementary Figure 1.** Performance of the rats on the acquisition days 1-5. Post-hoc tests showed no differences between the groups in the number of entrances (a), shocks (b) and time to the first entrance (d). However, the total path was significantly different on days 4 [p = 0.0006] and 5 [p = 0.0411] in the mPFC groups. On both days, the BI/mPFC group had lower locomotion than the UNI/mPFC group.

## Supplementary Table

| 1. **Unilateral / bilateral mPFC** | | | | |
| --- | --- | --- | --- | --- |
| **1) Sessions on days 1-5** | **Effect of session** | **Effect of group** | **Effect of interaction** | **Transformation** |
| Entrances | **F (4, 64) = 40.40, p < 0.0001** | F (1, 16) = 1.374, p = 0.2583 | F (4, 64) = 0.4138, p = 0.7981 | ln(y+1) |
| Shocks | **F (4, 64) = 54.63, p < 0.0001** | F (1, 16) = 2.463, p = 0.1361 | F (4, 64) = 0.09513, p = 0.9837 | ln(y+1) |
| Time to the first entrance | **F (4, 64) = 7.542, p <0.0001** | F (1, 16) = 1.352, p = 0.2620 | F (4, 64) = 1.325, p = 0.2702 | ln(y+1) |
| Total path | **F (4, 64) = 2.798, p = 0.0332** | **F (1, 16) = 10.77, p = 0.0047** | F (4, 64) = 1.638, p = 0.1755 | - |
| 1. **Unilateral / bilateral vHPC** | | | | |
| **1) Sessions on days 1-5** | **Effect of session** | **Effect of group** | **Effect of interaction** | **Transformation** |
| Entrances | **F (4, 60) = 39.02, p < 0.0001** | F (1, 15) = 0.01309, p = 0.9104 | F (4, 60) = 0.09079, p = 0.9850 | - |
| Shocks | **F (4, 60) = 48.66, p < 0.0001** | F (1, 15) = 0.04651, p = 0.8321 | F (4, 60) = 0.09781, p = 0.9828 | ln(y+1) |
| Time to the first entrance | **F (4, 60) = 4.258, p = 0.0042** | F (1, 15) = 1.121, p = 0.3064 | F (4, 60) = 0.8327, p =0.5097 | ln(y+1) |
| Total path | F (4, 60) = 1.516, p = 0.2090 | F (1, 15) = 0.2060, p = 0.6564 | F (4, 60) = 0.8805, p = 0.4811 | - |
| 1. **Contralateral / ipsilateral mPFC-vHPC** | | | | |
| **1) Sessions on days 1-5** | **Effect of session** | **Effect of group** | **Effect of interaction** | **Transformation** |
| Entrances | **F (4, 47) = 22.90, p < 0.0001** | F (1, 12) = 0.04233, p = 0.8404 | F (4, 47) = 1.186, p = 0.3292 | ln(y+1) |
| Shocks | **F (4, 47) = 32.10, p < 0.0001** | F (1, 12) = 0.02878, p = 0.8681 | F (4, 47) = 0.9262, p = 0.4568 | ln(y+1) |
| Time to the first entrance | **F (4, 47) = 9.674, p < 0.0001** | F (1, 12) = 0.007547, p = 0.9322 | F (4, 47) = 0.5421, p = 0.7056 | sqrt(y+0.1) |
| Total path | F (4, 47) = 2.320, p = 0.0706 | F (1, 12) = 0.04273, p = 0.8397 | F (4, 47) = 0.4845, p = 0.7470 | ln(y-35) |

**Supplementary Table 1.** Summary of two-way ANOVA (A, B) and mixed-effect models (C) results between the experimental groups on the acquisition days 1-5. Significant values are in bold.
